# The effect of observation angles on facial age perceptions: A case study of Japanese women

**DOI:** 10.1371/journal.pone.0279339

**Published:** 2022-12-27

**Authors:** Motonori Kurosumi, Koji Mizukoshi, Maya Hongo, Miyuki G. Kamachi

**Affiliations:** 1 Graduate School of Informatics, Kogakuin University, Shinjuku, Tokyo, Japan; 2 POLA Chemical Industries, Inc., Yokohama, Japan; 3 Faculty of Informatics, Kogakuin University, Shinjuku, Tokyo, Japan; Universidade Federal Fluminense, BRAZIL

## Abstract

Most conventional aging research has limited its approach concerning the head and face shape and skin condition to the frontal face. However, in our daily lives, we observe facial features from various angles, which may reveal or obscure aging features that could only be identified under limited conditions in the past. This study systematically investigates the effect of facial observation angles—specifically, of horizontal and vertical angles—on age impression. A total of 112 Japanese women aged 20–49 years participated as observers who evaluated the age impressions of 280 Japanese women aged 20–69 years. A two-way analysis of the variance of the age impression score was conducted for two factors: observation angle (five angles with yaw and pitch directions) and age group (five ages, from the 20s to the 60s). The results reveal that, as compared with frontal observation, the perceived age tended to decrease with the facial observation angles and that the effect of the angle on perceived age decreased with increasing age, especially for the profile face. Understanding the effect of the facial observation angle on age impression and clarifying the characteristics of the face and skin not perceived in the frontal face will provide useful knowledge to make people look youthful, look more beautiful, and be happier in all aspects of their lives.

## Introduction

The human face can be described as an interface in which a variety of social information is embedded and it is the starting point of interpersonal communication. Different persons can be identified by observing biological attributes, such as age, gender, and race, while social information, such as attractiveness, reliability, and competence, can be ascertained from their facial features. For instance, when we meet a person for the first time, the impressions we get from facial expressions have a great influence on our interpersonal evaluation [1]. Furthermore, a common desire is to remain youthful even as one ages. Youthfulness also plays a role in interpersonal attractiveness, and age impressions are sometimes more important than a person’s actual age.

Previous studies on the age impressions of facial appearance have reported the effects of face shape and color information [2]; of eyes, nose, and mouth placement and size [3]; and of skin morphological and tonal characteristics such as wrinkles, spots, and sagging [4–8]. Most of these studies have limited the appearances in question to frontal face images [2, 3, 6, 8, 9]. However, in real life, we observe faces from various angles, such as when we look at the profile of a driver from the passenger seat or up at the face of someone standing in front of us while sitting on a train. On social networking sites, many people post facial photos taken from above, and we know empirically that an angle can change the impression of facial appearance. Therefore, to elucidate the full extent of the age impressions we receive in real life, the effect of facial observation angles on age impressions should be examined.

In terms of face recognition, previous studies have reported that, in a matching task to determine whether two faces are the same person, the correct response rate was lower when frontal faces were compared with profile faces than when frontal faces were presented to each other [10, 11]. Conversely, it has also been reported that, where they have unfamiliar characteristics, oblique faces are easier to recognize than frontal [12].

As for the effect on facial impressions, although the facial angles themselves do not have a significant effect on impression formation, a relationship between these angles and expressions has been reported [13, 14]. For example, from a frontal angle, a smiling (happy) face appears to be more trustworthy when compared with oblique or profile faces. Additionally, downward or upward faces are perceived as negative expressions.

However, no studies have examined the effect of the facial observation angle on age impression. It is easily conceivable that differences in this angle can change the impression. Specifically, the size and direction of individual features, such as the eyes, nose, and mouth, as well as their arrangement, appear to change depending on the observation angle, which affects facial impression as a whole. Additionally, depending on the observation angle, the appearance of skin morphology, such as wrinkles and sagging, can be emphasized or obscured, which also contributes to a change in the age impression.

Understanding the effect of the facial observation angle on age impression and clarifying the characteristics of the face and skin not perceived frontally would provide useful knowledge for beauty. This knowledge can be used to make people look youthful, look more beautiful, and be happier in all aspects of their lives. For example, it is possible to correct any skin appearance with makeup and to prevent and improve facial aging characteristics related to the age impression through skin care and treatment.

To systematically understand the effect of facial observation angles on the apparent age impression, this study examined the effect of horizontal and vertical angles on perceived age. To verify the possibility that facial aging features change the perceived age depending on the observation angle, the effect of the angle for each age group of the face model was also examined. An age-perception test was conducted using Japanese women as face models, and observers were employed.

## Materials and methods

### Ethics statement

All demonstration tests related to this study were conducted according to the protocol approved by the ethical committee of POLA Chemical Industries, Co., Ltd., following the Declaration of Helsinki (Approval No. 2014-G-106; October 14, 2014, Approval No. 2014-G-139; December 5, 2014, Approval No. 2015-G-005; January 16, 2015, Approval No. 2015-G-023; February 27, 2015, Approval No. 2016-I-140; October 24, 2016). Written informed consent was received from all participants and face models to publish these case details.

### Participants

A total of 112 Japanese women aged 20–49 years participated as observers who evaluated age impressions. Observers did not have any background regarding the subject of study. The sample size was determined through calculations using G*Power 3.1.9.7 software [15]. In the absence of prior data, the G*Power default values were used for the current calculation, and power analysis indicated that 98 participants were needed for two-way analysis of variance, effect size *F* = 0.10, *α* = 0.05, and power = 0.90. The observers were younger than 50 years because a previous study reported that human vision’s perceptual function [16] and the brain’s information processing speed [17] decline as people age, especially among those in their 50s and older. Table 1 shows the age breakdown of the observers.

**Table 1:** Participant characteristics.

Age group(years)	N	Age, Mean (years)	Age, S.D. (years)
20–29	38	24.84	2.99
30–39	36	34.58	2.84
40–49	38	43.87	3.26

### Stimuli

The face models were Japanese women aged 20–69 years. All models were ordinary, paid subjects and unfamiliar to the observers. To eliminate the influence of individual differences in makeup, the models were asked to participate in this experiment without it.

Three-dimensional images of the models’ faces were captured in the sitting position with eyes open by a 3D imaging system (VECTRA M3, Canfield Technology, Fairfield, ND, USA). The head direction of face models was standardized so that the Frankfurt plane [18–20] connecting the inferior orbital margin and upper margin of the external auditory canal was horizontal to the ground surface. During this time, the face models were asked to fixate on the reference point on the front that was set beforehand so that their gaze on the frontal angle of the display coincided with the observer’s gaze.

To verify the effect of the facial observation angle on perceived age, face models with similar perceptual ages were selected in advance. In the preselection process, 280 face models were selected from 420 Japanese women using the following method. First, the models were divided into 10 age groups according to their actual age, in increments of five years. Twenty-eight people in each group with the least variation in perceived age from frontal faces were then selected using the age-perception test by 18 Japanese women. Consequently, each of the 10 age groups had 28 facial models. Table 2 shows the breakdown by age.

**Table 2:** Stimuli characteristics.

Age group (years)	N	Age, Mean (years)	Age, S.D. (years)
20–24	28	23.21	1.87
25–29	28	27.54	1.79
30–34	28	32.29	1.67
35–39	28	37.79	1.17
40–44	28	41.04	1.17
45–49	28	46.43	1.64
50–54	28	51.93	1.54
55–59	28	55.64	2.71
60–64	28	61.07	1.09
65–69	28	66.14	1.76

**Fig 1:**
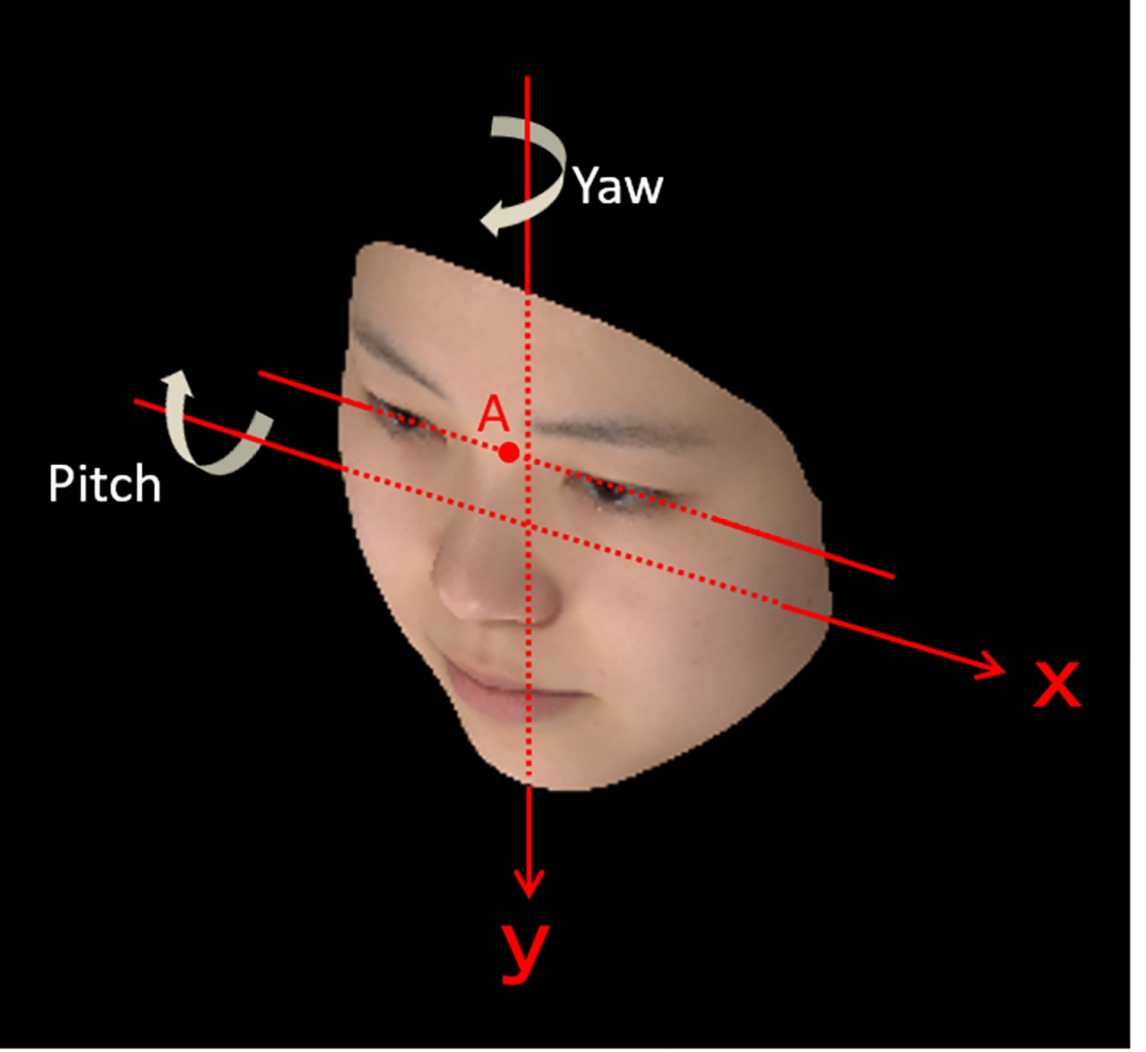
The location of landmark for facial orientation. To set the facial observation angle, the face image was rotated in the yaw and pitch directions with an origin at landmark A, which was set at the midpoint of the pupils. The photographs show an averaged face of models in their 20s as an example.

The faces of the models were normalized by (A) adjusting the orientation and position of the individual face, basing the pitch angle on the Frankfurt plane; (B) adjusting the yaw and roll angles according to the symmetry of the contour line image displayed on the frontal face view; (C) adjusting the spatial position as the origin of head rotation and aligning the midpoint of the upper ear canal for all the subjects’ faces; and (D) deleting the subjects’ hair and areas below the neck, which were unnecessary for the analysis. Processing was performed using face-shape analysis software (Face-Rugle, Medic Engineering, Inc., Kyoto, Japan). On this software, landmark A, the midpoint of the pupils of the eyes in Fig 1 was marked as the origin of the stimulus.

**Fig 2:**
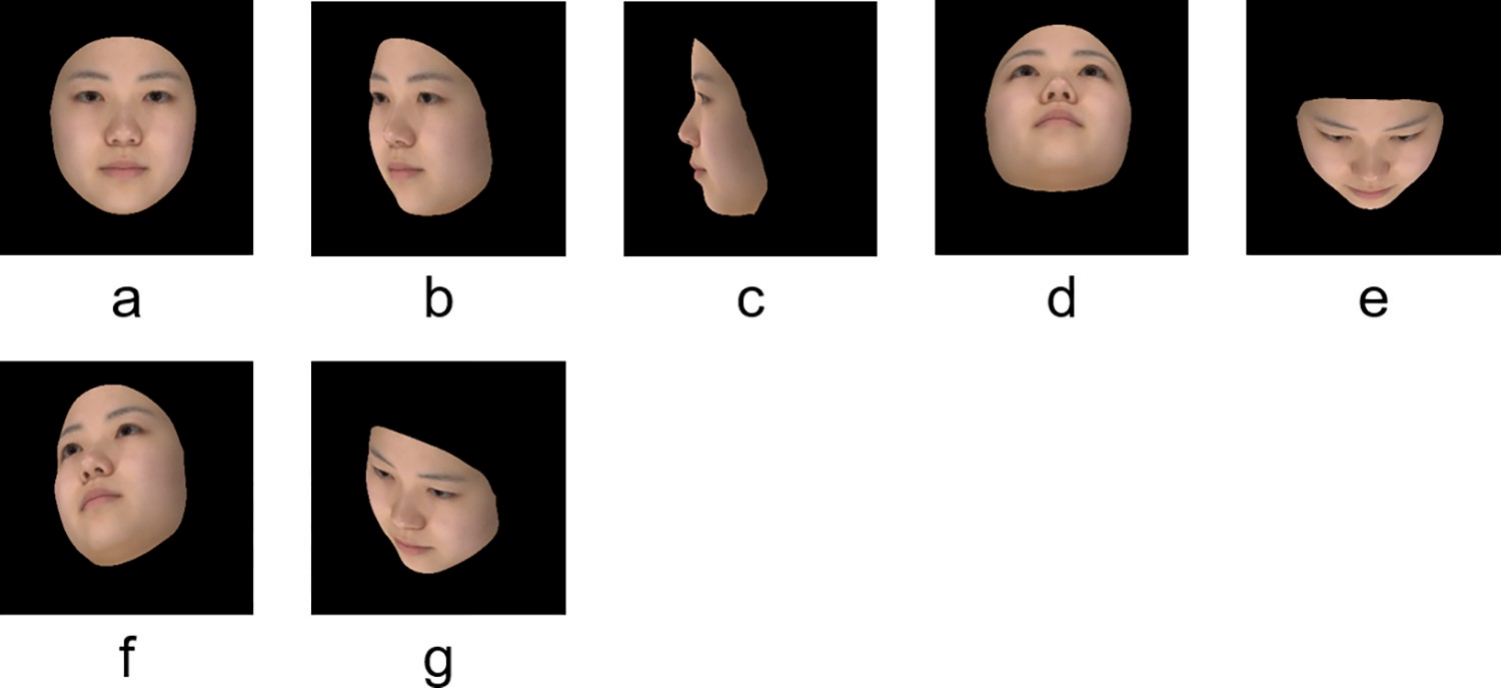
Face angle used as stimuli. Examples of face stimuli with angles: (a) 0° rotation as control and experimental stimuli; (b) 45° rotated right in the yaw, (c) 90° rotated right in the yaw, (d) 45°rotated pitch above, and (e) 45° rotated pitch below as experimental stimuli; (f) 33° rotated right in the yaw and pitch above and (g) 33° rotated right in the yaw and pitch below as dummy stimuli. The photographs show an averaged faces of models in their 20s as examples.

Next, five experimental stimuli, consisting of the frontal angle and the yaw or pitch angle, as well as two dummy stimuli, consisting of the yaw and pitch angle, were created. The dummy stimuli were used to prevent the observer from realizing the target “vertical” and “horizontal” direction, and responses were not recorded. “Frontal (a)” was used as the control and experimental stimuli and “45° rotated right in the yaw (b),” “90° rotated right in the yaw (c),” “45° rotated pitch above (d),” and “45° rotated pitch below (e)” as the experimental stimuli. Additionally, “33° rotated right in the yaw and pitch above (f)” and “33° rotated right in the yaw and pitch below (g)” were used as the dummy stimuli (Fig 2). In group conversations, opportunities arise to observe the horizontally angled face of a person seated next to us or for standing and seated people to observe each other’s faces from up and down directions, respectively. In this study, to verify the effects of the vertical and horizontal directions, the basic angle for the upper, lower, and side was 45 degrees, and, for the horizontal direction, five types of degrees were used, including the profile (90 degrees) that we may encounter in daily life.

The experimental stimuli (Fig 2(A)–2(E)) and dummy stimuli (Fig 2(F) and 2(G)) were presented for comparison with the control stimuli (Fig 2(A)). This study did not take into account the left–right difference but instead examined the yaw angle direction in one direction where the left side of the model’s face was watched by the observer. The face size was set as what it appears to be at a distance at which humans talk with each other daily. The face width (horizontal distance between the zygomatic arches) was adjusted to 12 cm (11.421° of visual angle) for each face model on a 24-inch color liquid-crystal display (Colour Edge CX2414, EIZO, Japan). The face stimuli were observed at a viewing distance of 60 cm.

### Procedure of age-perception test

**Fig 3:**
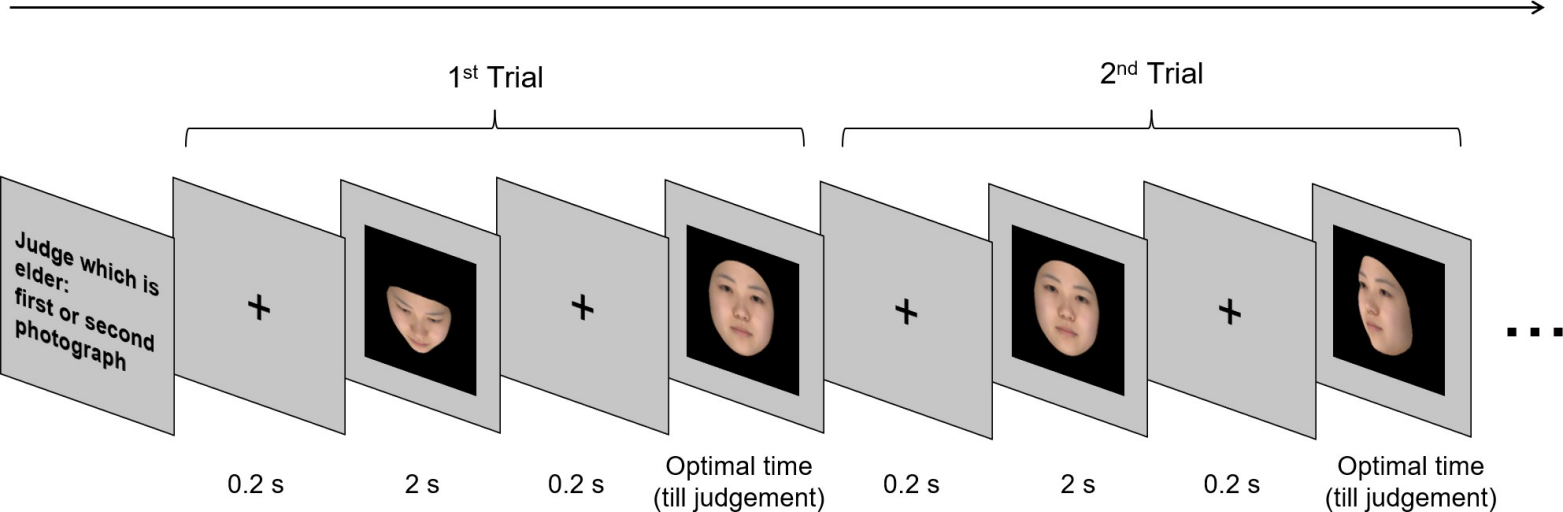
An example of the procedure of the experiment. After the presentation of instruction, a cross (+) indicating the fixation point was presented on a liquid-crystal display for 0.2 s, two faces were presented successively—the control stimulus and either an experimental or a dummy stimulus—for 2 s. After the presentation, the participant chose the face that appeared older by pressing a button. Whether the control stimulus appeared first or second was random and the number of presented stimuli was counterbalanced by observation angles. The observer evaluated the faces of all 280 models in 10 blocks with models’ age groups. The photographs show an averaged face of models in their 20s as examples.

After two faces were successively presented, the observer was asked to select, using a keyboard, the face that appeared older. One of the faces was of the frontal angle (control stimulus) and the other was either the experimental or the dummy stimulus. To investigate the effect of facial observation angles on age impression more precisely, the age-perception test was divided into 10 age groups, at increments of 5 years, with 28 participants in each group. A total of 112 observers performed trials for each age group. There were 14 trials in each age group, for a total of 140 trials. The observers evaluated each face model only once. The number of evaluation times of a facial observation angle was counterbalanced and the presentation order was randomized among observers. The presentation time of the face was set at 2 s for the first face presented and within 2 s until the age judgment was made for the second face presented (Fig 3). Data that took more than 2 s to evaluate for the faces were excluded.

For each of the five angles presented as the experimental stimuli, except for dummy stimuli, the percentage of faces judged to be older than the frontal face (control stimulus) as the age impression score was calculated for each age group of the models. A two-way analysis of variance to the age impression score was conducted for the observation angle (five angles) and the age group (five ages). All the statistical analyses were conducted using SPSS, version 25.0 (IBM, Armonk, NY, USA).

## Results

**Fig 4:**
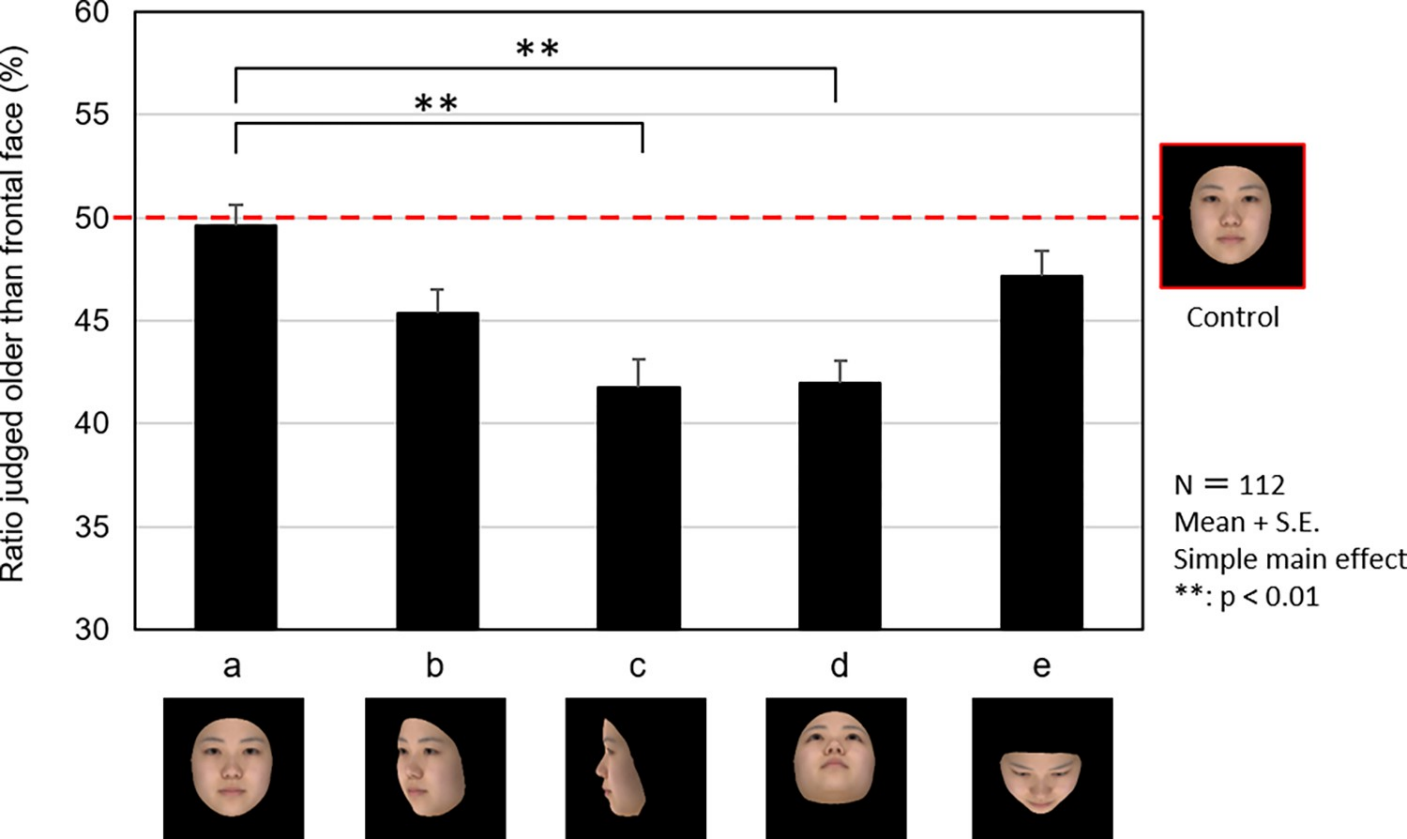
Effects of the facial orientation on age impression. The graph shows the percentages of 112 observers who judged experimental stimuli (frontal face (a) and angled face (b, c, d, and e)) as older than control stimuli (frontal face). The closed columns and the vertical lines on the columns are the mean values and standard errors, respectively. The photographs show an averaged face of models in their 20s as an example.

The results of the two-way analysis of variance on the age impression between the observation angle (five angles) and model age (five ages) revealed the effect of the former [*F* (4, 444) = 7.812, *p* < 0.001, *η*_*p*_^*2*^ = 0.066], confirming that age impression differs based on observation angle (Fig 4). The results of the multiple comparison test using Bonferroni’s method confirmed that the perceived age was significantly lower for a face observed from the side (*p* < 0.01) and one observed from below (*p* < 0.01) compared with frontal observation.

However, the effect of the observation angle was limited by an interaction with the age of the face model [*F* (16, 1776) = 1.746, *p* < 0.05, *η*_*p*_^*2*^ = 0.015]. The statistical values are shown in Table 3.

**Table 3:** Results of the two-way analysis of variance of age impression between observation angle and model age group.

Source	*F (dfb*, *dfw)*	*p*	*η*_*p*_^*2*^
Angle	7.812 (4,444)	< 0.001***	0.066
Age group	0.085 (4,444)	0.987	0.001
Angle × Age group	1.746 (16,1776)	0.033*	0.015

**Fig 5:**
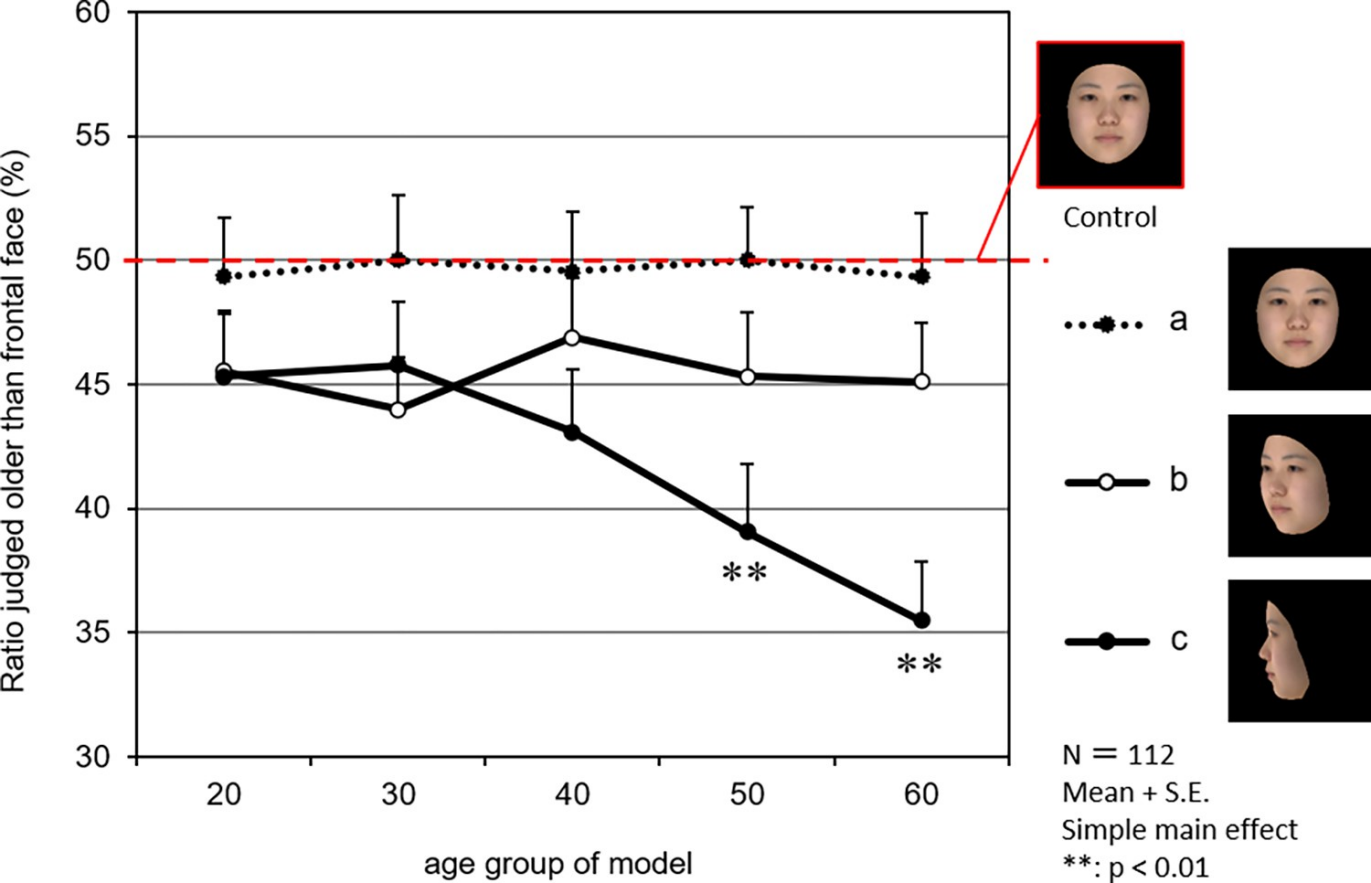
Effects of the model’s age group on age impression by facial yaw orientation. The graph shows the percentages of 112 observers who judged experimental stimuli (frontal face (a) and angled face (b, c)) as older than control stimuli (frontal face). The closed circles with dotted lines show the frontal face (a). The open circles with solid lines show the face 45° rotated right in the yaw (b). The closed circles with solid lines show the face 90° rotated right in the yaw (c). The marks and the vertical lines on the marks are the mean values and standard errors, respectively. The photographs show an averaged face of models in their 20s as an example.

Fig 5 shows the effect of the face model’s age and lateral orientation on age impressions. At 45° to the right, the age of the face model did not have any effect on perceived age. By contrast, at 90° to the right, the perceived age decreased as the age of the face model increased, and in the 50s (*p* < 0.01) and 60s (*p* < 0.01), the age impression decreased significantly when compared with the frontal face.

**Fig 6:**
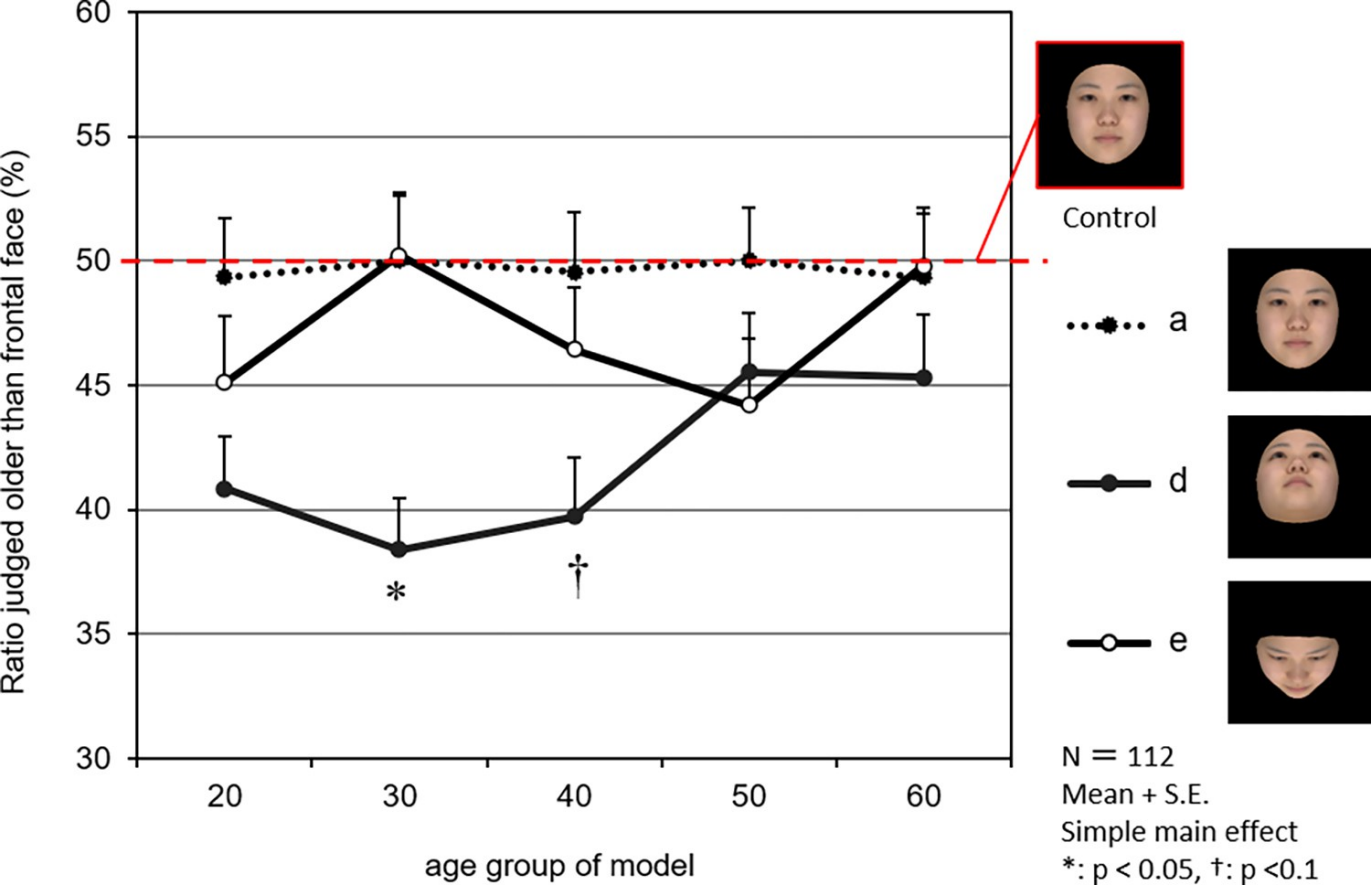
Effects of model’s age group on the age impression by a facial pitch orientation. The graph shows the percentages of 112 observers who judged experimental stimuli (frontal face (a) and angled face (d, e)) as older than control stimuli (frontal face). The closed circles with dotted lines show the frontal face (a) and the open circles with solid lines show the face at 45° rotated pitch above (d) and 45° rotated pitch below (e). The marks and the vertical lines on the marks are the mean values and standard errors, respectively. The photographs show an averaged face of models in their 20s as an example.

Next, Fig 6 shows the effect of the age of the face model on the age impression in vertical directions. In the faces observed from a downward direction, a tendency for the perceived age to decrease was confirmed for face models in their 30s or younger compared with those in their 50s or older, with a significant decrease in the 30s (*p* < 0.05) and a decreasing tendency in the 40s (*p* < 0.1). In faces observed from an upward direction, no effect of the age of the face model on the perceived age was confirmed.

## Discussion

In this study, an age-perception experiment was conducted using Japanese women as face models and observers, and the effect of the facial observation angle on age impressions was evaluated. The results revealed that angled faces tended to be judged as younger than frontal ones. Additionally, the effect of the observation angle on lowering the perceived age differed depending on the age of the face model.

Previous research on the age impression of facial appearance has reported the effects of the head shape; the arrangement of eyes, nose, and mouth; morphological features of the skin such as facial wrinkles and sagging; and tonal features such as spots and dullness, though mainly for frontal face images [2, 3, 6, 8, 9]. However, the visibility of facial features that affect age impressions can change depending on the daily scene. The results of this study revealed that, generally, non-frontal faces reduced the apparent age impression more than frontal.

However, the effect of the observation angle on age impression varied depending on the age of the face model. When the faces were observed from the side, the effect of decreasing the age impression was found to be greater as the angle at which the face was observed increased from 45° to 90° (Fig 4). When the age of the model was taken into account, the perceived age of the face was found to reduce as the age of the face model increased at 90° in a yaw angle of observation (Fig 5).

When the face is divided into three parts, from the midline to the outer cheek, signs of skin aging, such as wrinkles and sagging, become more pronounced in the central region where there is little subcutaneous support tissue [21]. Because this study’s subjects were Japanese, who have less chiseled faces than Whites, this area could hardly be observed at a 90° angle, which may have contributed to the reduced age impression. Moreover, research has shown that facial asymmetry increases with age [22–24]. The fact that this asymmetry cannot be evaluated in a face in full profile is also considered a factor in the decrease in perceived age.

As for the vertical observation angle, faces observed from above showed no significant reduction in age impression compared with frontal observation. By contrast, faces observed from below were found to have a significantly lower age impression than those observed frontally (Fig 4). When the age of the face model was taken into account (Fig 6), we confirmed that faces observed from below were more effective in reducing age impression when their models were young (e.g., in their 20s–40s). The effect of observation from below on perceived age was not observed in participants in their 50s and 60s, however.

The human face elongates longitudinally as the head skeleton grows [25]. From psychological experiments, the aspect ratio of the head and the size of the eyes are involved in the impression of infantilism [26, 27]. An angle of observation from below gives the impression that the aspect ratio of the face is shortened in the vertical direction compared with the frontal face. Additionally, the upper eyelids’ covering the eyes has less effect and the eyes appear larger. This is why the effect of the lower viewing angle on the perceived age was more pronounced in the younger age group than that in the older age group. Viewing a face from below increases the area occupied by the skin from the lower part of the eyes to the cheeks. This cheek area has macroscopic unevenness associated with skin aging, such as mid-cheek lines and sagging skin, which become more pronounced in women in their 50s and older [28, 29]. The appearance of this uneven structure in the cheek area may have weakened the effect of youthfulness because of the angle of observation.

However, this study had limitations. First, it focused only on the effect of the facial observation angle on age impression, and the lighting conditions were set in front of the faces for all angles. The lighting environment in daily life is sunlight or artificial lighting, where the conditions lighting the face change depending on the head orientation and various shadows may be produced because of diffuse and specular reflections as well as skin unevenness. A previous study found that changes in lighting conditions affect facial recognition [30–32]. Second, in this study, the faces were rotated in a virtual space with the gaze directed in front of it. In daily life, there may be situations in which the skin of the face is stretched by rigidly moving the head; the direction of gravity received by the skin is changed; or the observation angle is changed by the direction of the eyes, such as by looking upward. Future studies should thus consider the lighting conditions, the stretched skin or gravity conditions associated with the head orientation, and the direction of the gaze to better understand the age impressions we feel in our daily lives.

Third, this experiment used facial stimuli that did not include the hair or neck regions. Notably, hairstyles and wrinkles on the neck change depending on the observation angle, and age is mostly judged based on the overall impression created by all of these factors. Therefore, future studies should consider all the characteristics of the face and its surroundings, which change depending on the angle from which the face is observed.

Finally, this study used Japanese women as models and observers. In terms of facial models, Whites have more three-dimensional facial structures than Asians [33–38] and men have more chiseled faces than women [39]. It has also been reported that the impressions of faces [40, 41] and gaze [42] are different in different cultures and that there are gender differences in the formation of these impressions, such as emotion recognition [43–46]. Therefore, whether the present results apply to other cultures and genders should be examined.

## Conclusion

This study is the first to systematically investigate the effect of facial observation angles on age impression. Previous studies focused only on the age impression of the face and skin via the frontal face. The results here reveal elements of the face and skin that affect age impressions and cannot be ascertained from the frontal face. Compared with previous studies, the possibility revealed here, of visually apparent or subtle aging characteristics depending on observation angle, makes the following contributions. First, it provides beneficial knowledge for cosmetic and image processing purposes and contributes to the development of solutions to make people look younger and more attractive. Second, it enables a more accurate and robust evaluation of the human condition in situations where facial appearance is evaluated by sensors for medical examinations and health maintenance. Future studies should, however, examine the universality of our results by expanding the scope of our study to men and people in other cultures.
